# Multidrug-Resistant Tuberculosis Treatment Outcomes in Karakalpakstan, Uzbekistan: Treatment Complexity and XDR-TB among Treatment Failures

**DOI:** 10.1371/journal.pone.0001126

**Published:** 2007-11-07

**Authors:** Helen S. Cox, Stobdan Kalon, Sholpan Allamuratova, Vinciane Sizaire, Zinaida N. Tigay, Sabine Rüsch-Gerdes, Hamraev A. Karimovich, Yared Kebede, Clair Mills

**Affiliations:** 1 Macfarlane Burnet Institute for Medical Research and Public Health, Melbourne, Australia; 2 Médecins Sans Frontières, Tashkent, Uzbekistan; 3 Médecins Sans Frontières, London, United Kingdom; 4 Ministry of Health, Karakalpakstan, Uzbekistan; 5 Forchungszentrum Borstel, National Reference Center for Mycobacteria, Borstel, Germany; 6 Médecins Sans Frontières, Amsterdam, The Netherlands; Duke University, United States of America

## Abstract

**Background:**

A pilot programme to treat multidrug-resistant TB (MDR-TB) was implemented in Karakalpakstan, Uzbekistan in 2003. This region has particularly high levels of MDR-TB, with 13% and 40% among new and previously treated cases, respectively.

**Methodology:**

This study describes the treatment process and outcomes for the first cohort of patients enrolled in the programme, between October 2003 and January 2005. Confirmed MDR-TB cases were treated with an individualised, second-line drug regimen based on drug susceptibility test results, while suspected MDR-TB cases were treated with a standardised regimen pending susceptibility results.

**Principal Findings:**

Of 108 MDR-TB patients, 87 were started on treatment during the study period. Of these, 33 (38%) were infected with strains resistant to at least one second-line drug at baseline, but none had initial ofloxacin resistance. Treatment was successful for 54 (62%) patients, with 13 (15%) dying during treatment, 12 (14%) defaulting and 8 (8%) failing treatment. Poor clinical condition and baseline second-line resistance contributed to treatment failure or death. Treatment regimens were changed in 71 (82%) patients due to severe adverse events or drug resistance. Adverse events were most commonly attributed to cycloserine, ethionamide and *p*-aminosalicylic acid. Extensively drug resistant TB (XDR-TB) was found among 4 of the 6 patients who failed treatment and were still alive in November 2006.

**Conclusions:**

While acceptable treatment success was achieved, the complexity of treatment and the development of XDR-TB among treatment failures are important issues to be addressed when considering scaling up MDR-TB treatment.

## Introduction

Since 1997, when the first global surveillance of tuberculosis (TB) drug resistance was reported, data on the threat that drug-resistant TB poses to TB control internationally have been growing [1], [2], [3]. Multidrug-resistant TB (MDR-TB), defined as resistance to the two most important anti-TB drugs isoniazid and rifampicin, has been found in all regions surveyed and is at critical levels in some areas, particularly among countries of the former Soviet Union.

MDR-TB is significantly more difficult to treat than drug-susceptible TB, requiring the use of less effective second-line drugs, which are often associated with major side effects. In response to the high cost of second-line drugs and the lack of treatment options for patients infected with MDR-TB, the World Health Organization (WHO), along with other partners, launched the Green Light Committee (GLC) in 2000 [4]. The aim of the GLC is to facilitate the treatment of patients with MDR-TB through extending existing directly observed treatment, short-course (DOTS) programmes for TB treatment, termed DOTS-Plus. Support includes the provision of technical support and preferentially priced drugs.

By the end of 2006, the GLC had endorsed 53 MDR-TB control projects, encompassing the treatment of more than 25,000 patients [5]. Results from 5 of these projects have been published, with treatment success ranging from 59% in the Philippines to 83% in the Russian Federation [5]. These results are in general a considerable improvement over those reported prior to the development of the DOTS-Plus programmatic approach [6]. However, despite the large numbers approved for MDR-TB treatment, the feasibility of scaling up MDR-TB treatment to cover all patients in need has not been demonstrated in resource-limited settings. This is significant in light of recent calls to scale up MDR-TB treatment to avoid the development of what is now classified as extensively drug-resistant TB (XDR-TB). XDR-TB, defined as MDR-TB with additional resistance to a fluoroquinolone and a second-line injectable agent, is often considered untreatable and was responsible for a well-publicised outbreak in South Africa [7].

Karakalpakstan is a semiautonomous region in the west of Uzbekistan, characterized by poverty, severe environmental degradation and slow reform of health services [8], [9]. In response to an identified need, the medical aid agency Médecins Sans Frontières (MSF), in cooperation with the Ministry of Health, began the progressive implementation of a DOTS program for TB treatment in 1998. By 2003, DOTS implementation was complete, covering a population of around 1.2 million, with a case notification rate of 482/100,000/year for all forms of TB [10]. However, poor treatment outcomes prompted a survey of TB drug resistance in 2001–2002, which found extremely high rates of MDR-TB; 13% among new cases and 40% among TB patients who had received prior TB treatment [10]. To date, these figures are the only data on the prevalence of MDR-TB in Uzbekistan.

These high levels, in turn, prompted the initiation of a pilot DOTS-Plus project to provide treatment for patients infected with drug-resistant TB strains in two of the 17 districts in Karakalpakstan. The program was started in the civilian sector in the main city of Nukus (population 260,600) and a rural district, Chimbay (population 97,700), some 50–100 km from Nukus. The aim was to assess the feasibility of DOTS-Plus in both the relatively accessible city and a rural area with low population density, poor transport and large distances. These districts had functioning DOTS programs for at least 2–3 years prior to the implementation of DOTS-Plus. The DOTS program utilised standard first-line treatment regimens, along with direct sputum smear microscopy for diagnosis, as recommended by WHO [11]. Despite the adoption of DOTS, both first- and second-line anti-TB drugs are available for private sale in Karakalpakstan.

The pilot DOTS-Plus project received GLC approval to treat 100 patients in early 2003, with the first patient starting treatment in October 2003. In late 2004, a further GLC application was submitted and approval was given to enrol up to 800 patients on treatment. As of June 2007, 433 patients have been registered in the program. Here we report treatment outcomes for the first cohort of patients treated in the DOTS-Plus pilot project in Karakalpakstan, Uzbekistan and the risk of XDR-TB among treatment failures.

## Materials and Methods

### Setting and study design

Since this pilot program had initial approval to treat only 100 patients, the program was implemented separately from the DOTS program. This was based on the realisation that not all patients with MDR-TB would be able to be treated. For inpatient care of patients with MDR-TB, an unused hospital on the outskirts of Nukus City was reorganized in 2003 to house 50 patients and has since been upgraded to 75 beds. A separate outpatient clinic was established in Nukus City, and existing DOTS clinics were utilized to provide ambulatory services for DOTS-Plus in Chimbay district. In addition, a laboratory capable of culture and drug susceptibility testing (DST) was established in Nukus. The pilot program is a collaborative effort between MSF, the Ministries of Health in Karakalpakstan and Uzbekistan and the National Reference Centre for Mycobacteria in Germany.

**Figure 1:**
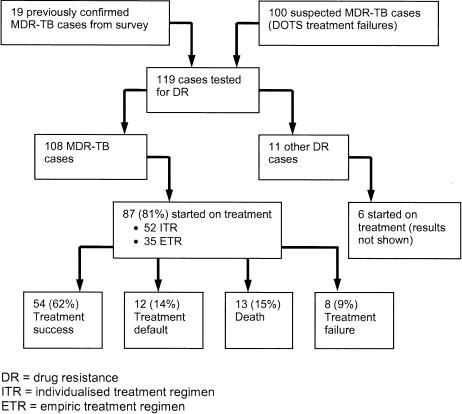
Patient recruitment and outcomes from MDR-TB treatment October 2003 to January 2005.

The first cohort of patients to be enrolled in the DOTS-Plus pilot project consisted of: patients found to be infected with MDR-TB during the initial drug resistance survey [10] and alive at the time of first enrolment (confirmed MDR-TB), or recorded as having previously failed a standard category-2 re-treatment DOTS regimen (suspected MDR-TB) (Figure 1). Suspected MDR-TB patients were either started on an empiric treatment regimen (ETR) or were placed on a waiting list until DST results became available. This decision was made based on clinical condition and hospital capacity. All patients were actively sought and further sputum samples obtained, with informed consent, prior to enrolment.

Patients were eligible for treatment if they were: residents of the two districts, Nukus City and Chimbay; had culture positive pulmonary TB; had previously been treated in the DOTS program; and did not have any of the conditions listed in the exclusion criteria and following assessment by a treatment committee established for the DOTS-Plus program. Exclusion criteria were listed as any concomitant medical conditions that in principle precluded anti-TB treatment. These are described as cirrhosis, uncontrolled seizure disorder, significant psychiatric disease and known allergies to second-line anti-TB drugs. Generally, poor clinical condition was not an exclusion criterion. Patients were also not excluded based on prior default from TB treatment. For the first 2 years of the program, only adult patients (aged over 16 years) were eligible for treatment. Since then, children are now eligible for home-based treatment. Although only patients from the two districts, Nukus and Chimbay, were eligible for enrolment, a further two patients with MDR-TB from the drug-resistance survey who resided in a separate rural district were found to still be alive at the time of enrolment and were also included.

A small number of suspected MDR-TB patients were found to be infected with polyresistant strains and were treated accordingly (Figure 1); they have not been included in this analysis, which includes only MDR-TB patients enrolled between October 2003 and January 2005. All patients received a detailed explanation of the treatment protocol and signed a written consent form prior to treatment. As this was a programmatic evaluation and all data were collected routinely, ethical approval was not sought prior to the implementation of the program nor this evaluation.

### Treatment regimens and protocol

Suspected MDR-TB patients were started on an ETR consisting of pyrazinamide, ofloxacin, ethionamide, *p*-aminosalicylic acid (PAS), cycloserine and either capreomycin or kanamycin, depending upon reported previous use of kanamycin. Confirmed MDR-TB patients were started on an individualised treatment regimen (ITR) based on the most recent DST profile for that patient. Both empiric and individualised treatment regimens were adjusted when more recent DST results became available. In general, regimens contained at least five drugs to which the infecting strain was susceptible, including a second-line injectable agent (either capreomycin or kanamycin) for at least 6 months after documented sputum culture conversion. The total treatment duration included a minimum of 18 months of treatment after culture conversion. Culture conversion was defined as at least 2 negative cultures at least 30 days apart. The duration of hospitalisation was variable, depending on the use of an injectable drug in the regimen, clinical condition and availability of family support. In general, patients were hospitalised for the first 6 months of their treatment. Patients were not tested for HIV at any stage during treatment.

Sputum smear and culture were conducted monthly, and, when culture positive, DST was reassessed at 4 months of treatment, or if a patient was culture positive after previously being culture negative. Chest radiographs were performed at enrolment, after 3 months of treatment and every 6 months thereafter. Adverse events were managed rapidly and aggressively, with permanent removal of a drug from the treatment regimen as a last resort. All treatment doses of drugs were directly observed, with the exception of amoxicillin/clavulanate for discharged patients, where doses were divided across the day, with only the first half-dose observed. Resectional surgery was not undertaken for any of the patients in this first cohort. Patients received counselling to maximise adherence and financial support for transport to outpatient facilities when discharged from hospital. During hospitalisation, at least four meals were provided daily, and the families of patients received a food parcel on a monthly basis while the patient was still receiving treatment.

### Definitions

Treatment outcomes were defined according to recommendations from the WHO MDR-TB working group [12]. Cure was defined as at least five negative sputum cultures in the last 12 months of treatment. A single positive culture was allowed if it was followed by a minimum of three negative cultures. Patients were classified as having completed treatment if there were insufficient bacteriological results to classify the patient as cured, but no evidence of treatment failure. Treatment failure was defined as two or more positive cultures in the last 12 months of treatment, or if a medical decision was made to terminate treatment due to poor response or adverse events. Default was defined as an interruption of two or more consecutive months to treatment. Patients were recorded as dead if they died during treatment, regardless of the cause.

Severe clinical condition at treatment initiation was defined as one or more of the following: ability to walk unaided, resting respiratory rate ≥30/min or body mass index (BMI) <16 kg/m^2^. XDR-TB was defined as MDR-TB with the addition of resistance to a fluoroquinolone and to either an aminoglycoside or capreomycin or both [13]. Serious adverse events were defined as those that resulted in any change to the anti-TB drug regimen, either changing the dose of a drug, or temporarily or permanently removing a drug from the regimen.

### Laboratory testing

Sputum smear microscopy, culture and DST were conducted according to international standards in the Nukus mycobacteriology laboratory. Smears were assessed using fluorescence microscopy and culture using Lowenstein-Jensen media. All cultures were also sent from Nukus to the supranational reference laboratory in Borstel, Germany for repeat DST. For this cohort, all DST results reported were from the Borstel laboratory. DST was conducted for 5 first-line drugs (isoniazid, rifampicin, ethambutol, streptomycin, pyrazinamide) and 6 second-line drugs (capreomycin, amikacin, ofloxacin, ethionamide, cycloserine, PAS), as described previously [14].

### Data collection and analysis

A paper-based and computerized medical record system was instituted from the start of the pilot program. A system of data collection forms for enrolment and treatment follow-up were developed and the data entered routinely into an Epi-info-based database created specifically for the program (Epi-info version 6.04, CDC, Atlanta, GA). This system was designed to record significantly more information than that routinely collected in the DOTS program, where patient data from the entire treatment course were recorded on a single, two-sided treatment card.

During this analysis, a few data points not routinely recorded on the database were found to be important and were retrospectively compiled from the paper-based medical record. These included resting respiratory rate and ability to walk unaided at admission. These changes will be incorporated into a new database system for MDR-TB treatment currently under development.

Analyses were performed using either Epi-Info or SPSS (SPSS version 10.0, SPSS Inc., Chicago, IL) after exporting the data from Epi-Info. Differences in proportions were assessed using Pearson's Chi^2^ test, with *p*<0.05 considered significant. Multiple logistic regression analyses were used to assess the association between patient factors and poor treatment outcomes. Two logistic regression analyses were conducted: the first comparing treatment success with death or treatment failure (defaulters removed); and the second comparing treatment success with treatment default (deaths and failures removed). All the factors considered in the univariate analyses were entered into the multivariate analyses. These factors were based on existing literature and suggestions from the medical team involved in patient care.

## Results

### Case finding

Between October 2003 and January 2005, 119 patients underwent testing for drug resistance (Figure 1); 19 were patients previously found to be infected with MDR-TB in the drug resistance survey in 2001–2002, and 100 were suspected to have MDR-TB based on previously failing a category-2 DOTS regimen. At that time, DST was not routinely conducted in the DOTS program in the two pilot districts, and only patients who could potentially be offered treatment in the DOTS-Plus pilot underwent DST. The aim was to have as few people as possible waiting for admission at any point in time. Of the 119 patients, 108 (91%) were subsequently found to have MDR-TB (including all the 19 cases from the drug resistance survey). Therefore, among the 100 suspected MDR-TB cases, 89 (89%) were found to be infected with MDR-TB; the remaining 11 were all infected with strains with some level of drug resistance.

In total, 93 patients were started on treatment in the program; this included 6 other drug-resistant cases (treatment outcomes not shown). Of the 108 MDR-TB cases, 87 (81%) were started on treatment. Of the remainder, the reasons for not starting treatment included death before admission (11 patients, 10%), refusal to be treated (8 patients, 7%), moved out of the district (1 patient, 1%) and concomitant psychiatric disease precluding TB treatment (1 patient, 1%). While there were no significant differences in drug resistance profile between MDR-TB patients starting treatment and those not, those treated tended to have a greater level of pre-existing second-line drug resistance. Treatment outcome results are reported for the 87 patients in the cohort who started MDR-TB treatment.

### Baseline patient characteristics

Patients treated in this first cohort are described in Table 1. Median age was 34 years, ranging from 17 to 72 years; 61% were male. Patients had been ill with TB for a median of 4 years and had received a median of four previous TB treatment episodes. Overall, 44% of patients were classified as being in poor clinical condition, and 70% had bilateral cavitary disease.

**Table 1:** Description of 87 MDR-TB patients started on treatment

Patient characteristics	No. of patients (%) N = 87	Median (range)
*Demographics*		
Male sex	53 (61)	
Age		34 (17–72)
Rural residence	34 (39)	
Previous imprisonment	15 (17)	
Injection drug use (at admission)	2 (2)	
Excessive alcohol use (at admission)	20 (23)	
Tobacco use (at admission)	27 (31)	
Receiving a pension	65 (75)	
Health care worker	8 (9)	
*Previous TB treatment*		
Duration of TB disease (years)		4.1 (0.5–34)
Previous TB treatment episodes		4 (2–30)
Previous lung surgery	3 (3)	
Previous use of at least one second-line drug	57 (66)	
*Baseline clinical characteristics*		
Severe clinical condition*	38 (44)	
Hemoptysis	30 (34)	
Bilateral cavitary disease	61 (70)	
Body mass index (BMI)		17.4 (11.7–28.0)

* Defined as one or more of the following: inability to walk unaided, high resting respiratory rate (above 30/min) or BMI <16.0.

All patients had undergone a DOTS category-2 treatment regimen at least once, and 90% of patients had failed a category-2 regimen at least once. Although all patients had been treated within the DOTS program, 66% had also taken at least one second-line drug indicating previous treatment outside of the DOTS system. This drug was most commonly kanamycin, which is widely available in Karakalpakstan.

**Figure 2:**
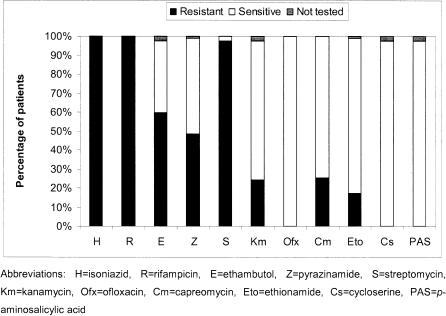
Drug resistance among 87 MDR-TB patients started on treatment.

The baseline resistance patterns are shown in Figure 2. High levels of first-line resistance were observed, with 31 (36%) of 87 strains resistant to all five first-line drugs tested. Apart from isoniazid and rifampicin, to which all strains were resistant, 85 (98%) of 87 were resistant to streptomycin, 52 (61%) of 85 were resistant to ethambutol and 42 (49%) of 86 were resistant to pyrazinamide.

Overall, 33 strains (38%) were resistant to at least one second-line anti-TB drug. The most common second-line resistance was to capreomycin (22/87, 25%) or kanamycin (21/85, 25%), followed by ethionamide (15/86, 17%). No resistance to ofloxacin, ethionamide or PAS was observed at baseline. Significant overlap between capreomycin and kanamycin resistance was seen; all 21 strains with resistance to kanamycin were also resistant to capreomycin, with one additional strain showing capreomycin resistance, but not kanamycin. No patients were infected with XDR-TB strains at the start of treatment, due to the absence of resistance to ofloxacin.

### MDR-TB treatment regimens

The majority of patients (52, 60%) were started on an individualised regimen, based on known DST results. The remaining 35 patients were treated with the empiric regimen, with DST results available a median of 61 days (range 6–182 days) after treatment initiation. For the majority of patients (25/35, 71%) started empirically, the receipt of DST results did not cause any change to the regimen. For the 10 remaining patients, the most common change to the regimen upon receiving DST results was the removal of pyrazinamide, based on measured resistance.

Patients were started on a regimen containing a median of six drugs (range 5–7) but were treated in total with a median of seven drugs (range 5–10). This discrepancy reflects the 71 patients (82%) requiring the composition of their treatment regimens to be changed at least once during the treatment course (Table 2). The most common drugs added to treatment regimens after treatment initiation were ethambutol (not included in the ETR and where susceptibility was later demonstrated on DST) and amoxicillin/clavulanate (added as an additional agent when more efficacious second-line drugs were no longer usable). The two most common reasons for stopping drugs in the regimen were the development of resistance and severe adverse events (resulting in the permanent removal of the drug from the regimen) (Table 3). Resistance, either pre-existing or developed during treatment, was a significant cause of stopping the first-line drugs included in the regimen, with ethambutol and pyrazinamide stopped in about 40% of cases. In contrast, the development of serious adverse events presumed to be linked to particular drugs was most commonly responsible for stopping second-line drugs, in particular ethionamide, cycloserine and PAS. For a number of drug regimen changes, a reason was not recorded in the system (Table 3).

**Table 2:** Numbers of patients treated with individual drugs, in the starting regimen, added later, and stopped during treatment

	Started in initial regimen	Added later	Total given drug (% of total patients)	Stopped during treatment*(% of total given)
***First-line***				
H	0	0	0	0
R	1	1	2 (2%)	0
E	11	19	30 (34%)	14 (47%)
Z	68	3	71 (82%)	34 (48%)
S	0	2	2 (2%)	2 (100%)
***Second-line***				
Km	19	6	25 (29%)	2 (8%)
Cm	68	2	70 (80%)	8 (11%)
Ofx	86	1	87 (100%)	5 (6%)
Eto	79	2	81 (93%)	31 (38%)
Cs	83	4	87 (100%)	13 (15%)
PAS	84	2	86 (99%)	21 (24%)
Amx/Clv	14	38	52 (60%)	8 (15%)
Clz	3	11	14 (16%)	7 (50%)

* excluding drugs stopped as scheduled in the regimen, eg, the injectable agent (capreomycin or kanamycin).

Abbreviations: H = isoniazid, R = rifampicin, E = ethambutol, Z = pyrazinamide, S = streptomycin, Km = kanamycin, Cm = capreomycin, Ofx = ofloxacin, Eto = ethionamide, Cs = cycloserine, PAS = *p*-aminosalicylic acid, Amx/Clv = amoxicillin/clavulanate, Clz = clofazamine

**Table 3:** Reasons for permanently stopping individual drugs during MDR-TB treatment

Drugs given	No. patients given drug	No. stopped due to resistance (% of given)	No. stopped due to adverse events (% of given)	No. stopped due to other or unknown reasons (% of given)
E	30	11 (37%)	3 (10%)	0
Z	71	28 (40%)	4 (14%)	2
S	2	1 (50%)	1 (50%)	0
Km	25	0	2 (8%)	0
Cm	70	3 (4%)	3 (4%)	2
Ofx	87	5 (6%)	0	0
Eto	81	11 (14%)	19 (23%)	1
Cs	87	0	12 (14%)	1
PAS	86	0	20 (23%)	1
Amx/Clv	52	1 (2%)	2 (4%)	5
Clz	14	0	4 (29%)	3

Abbreviations: E = ethambutol, Z = pyrazinamide, S = streptomycin, Km = kanamycin, Cm = capreomycin, Ofx = ofloxacin, Eto = ethionamide, Cs = cycloserine, PAS = *p*-aminosalicylic acid, Amx/Clv = amoxicillin/clavulanate, Clz = clofazamine

Overall, 276 serious adverse events that resulted in a change to one or more anti-TB drugs in the regimen were recorded among 67 patients (67/87, 77%). A wide range of adverse events were attributed to the anti-TB drug regimen, including gastrointestinal disturbances (32% of adverse events) and neuropathies (13%). However, 54 (20%) did not have a clinical condition assigned. Similarly, assigning particular drugs as responsible for adverse events was often difficult; for 40 (14%) adverse events, multiple drugs were recorded as potentially responsible. The drugs most commonly held responsible were cylcoserine, ethionamide and PAS. Among the 80 adverse events attributed solely to cycloserine, 32 (40%) were described as neuropathies, 22 (28%) psychoses and 10 (13%) acute depression. For ethionamide and PAS, 30 (71%) of 42 and 28 (70%) of 40 adverse events, respectively, were described as gastrointestinal. These adverse events occurred throughout treatment, at a median of 7 months into treatment for cycloserine (range 1–20 months), 8 months (range 2–20) for ethionamide and 11 months (range 1–21) for PAS.

### Treatment outcomes

Treatment outcomes for the 87 patients started on treatment are given in Table 4. Overall, 54 (62%) of 87 patients were recorded as being successfully treated (cured and treatment completed) at the end of treatment, 12 (14%) did not finish treatment, 13 (15%) died during treatment and 8 (9%) were classified as treatment failures. While little difference was observed in treatment success among patients who were previously treated with second-line drugs, compared with those only treated with first-line drugs, a significant difference in the proportion defaulting treatment was seen: 33% of those treated only with first-line drugs, compared with 4% of those who reported taking a second-line drug for at least a month (*p* = 0.0001).

**Table 4:** MDR-TB treatment outcomes by previous treatment with second-line drugs

Outcome	Previously treated with first-line drug only	Previously treated with second-line drugs	Total
Cured	9	23	32
Completed	8	14	22
*Treatment success*	*17 (57%)*	*37 (65%)*	*54 (62%)*
Default	10 (33%)	2 (4%)	12 (14%)
Died	2 (7%)	11 (19%)	13 (15%)
Failure	1 (3%)	7 (12%)	8 (9%)
Total	30	57	87

Among the 12 patients recorded as treatment defaults, the median time to default was 6 months (range <1–17 months). Of the five patients who stopped treatment within the first 3 months, none had converted to sputum culture negative at the time of default. Among the remaining seven patients, six were culture negative at the time of default; including one patient who was culture negative for 13 months of treatment. One patient was still culture positive at 6 months of treatment when they defaulted. While extensive efforts were made to convince patients to restart treatment (both immediately after patients stopped taking treatment and after the 2-month period that defines default), only one of the 12 patients was restarted on treatment, but unfortunately defaulted from treatment again.

Of the 13 patients who died, only one died within the first month of starting treatment. The remainder survived for a median of 11 months of treatment (range 5–20 months). All but two of these patients were classified as being in severe clinical condition at treatment initiation, and two had undergone prior surgical resection of the lung as part of previous TB treatment. TB was considered by the treating physician to be an immediate or directly contributing cause of death in 12 of the 13 deaths; the remaining patient was a victim of homicide after 8 months of culture negativity. Among the eight patients classified as treatment failure, one was removed from treatment after 5 days due to a previously unrecognised concomitant illness that precluded treatment, while the remainder were removed from treatment due to failure of therapy after a median of 15 months on treatment (range 10–24 months). Of these, one patient died 4 months after stopping treatment, while the remaining six were still alive as of November 2006. These six patients are further discussed below.

### Treatment duration

Among successfully treated patients, the median total treatment duration was 22 months (range 18–30 months), which was a median of 18 months after culture conversion (range 16–23 months). Only one patient was treated for less than 18 months after culture conversion, as this patient became culture positive again after 8 months of initial culture negativity. For this patient, 16 months of culture negativity with a total of 31 months of treatment was considered sufficient. Lasting culture conversion among successfully treated patients was achieved in a median of 3 months (range 1–15 months) and the injectable was given for a median of 10 months after culture conversion (range <1–20 months), giving a total median duration for the injectable of 13 months among successfully treated patients (range 6–24 months). For some patients, the injectable could not be continued due to adverse events or drug resistance, and for others, the injectable could not be stopped due to concern about the total number of effective drugs remaining in the regimen. All patients classified as failing treatment were continued on the injectable for the duration of treatment.

### Treatment interruptions

Treatment interruptions, in which all drugs were stopped for a period of time, were due to adverse events, poor patient compliance, or both. Overall, interruptions to treatment were recorded for 84% of patients (73/87), resulting in a median of 18 days of treatment interruption per patient (range 0–117 days). Among successfully treated patients, a median of five separate interruptions occurred throughout treatment (range 0–17), encompassing a median duration of 17 days (range 0–85 days). This was not significantly different than the median of seven interruptions among the treatment-failure patients, with a median of 25 days of total treatment interruption.

### Factors associated with poor outcomes

Factors that might be expected to have a potential impact on treatment success, death or treatment failure, and treatment default are shown by univariate analysis in Table 5. Since the factors contributing to poor treatment outcomes are likely to be different for the outcome of treatment default compared with the outcomes of death or treatment failure, these outcomes have been analysed separately. Resistance to second-line drugs and severe clinical condition at treatment initiation were the only factors significantly associated with the outcomes of death or failure in the univariate analysis. Treatment interruptions, either ≥4 events or ≥30 days total were not significantly associated with death or failure on univariate analysis. In contrast, prior alcohol use (self-reported), the presence of hemoptysis at admission and *not* having taken second-line drugs previously were significantly linked to default on univariate analysis.

**Table 5:** Univariate analysis of factors potentially contributing to the outcomes of death and failure (combined) and default.

Factor	Treatment success (n = 54) %	Death or failure (n = 20) %	*p* value	Default from treatment (n = 13) %	*p* value
*Baseline characteristics*					
Male sex	61	55	0.79	69	0.75
Age ≥40 years	24	40	0.25	46	0.17
Previous imprisonment	20	10	0.49	15	1.0
Excessive alcohol use (self-reported)	19	15	1.0	54	0.034*
≥4 previous TB treatment episodes	57	70	0.42	62	1.0
Previous use of second-line drugs	69	90	0.076	15	0.001*
≥4 years of TB disease	48	60	0.44	54	0.77
Rural residence	48	25	0.11	23	0.13
Resistance to second-line drugs (any)	28	70	0.01*	31	1.0
Severe clinical condition	54	90	0.006*	77	0.21
Bilateral cavitary disease	72	80	0.57	46	0.10
Hemoptysis	26	40	0.26	62	0.022*
*Treatment characteristics*					
Started on empiric treatment regimen	35	40	0.79	62	0.12
≥30 days of treatment interruption	21	40	0.14		
≥4 treatment interruptions	59	65	0.79	39	0.22

* *p*<0.05. P values compare treatment success to death or failure, and to default, respectively.

In the multivariate analysis, severe clinical condition (OR 8.9, 1.6-50) and second-line resistance (OR 5.9, 1.6-22) remained the only significant predictors of death or failure. Prior heavy use of alcohol (OR 63, 2.8-1456) and severe clinical condition (OR 49, 1.7-1442) at the start of treatment were significant predictors of default, with previous use of second-line drugs (OR 0.01, 0.001-0.25) also remaining as a significant factor mitigating against default in the multivariate model.

### XDR-TB among treatment failures

The failure to effectively treat a significant number of patients despite available second-line drugs is concerning. To assess the risk posed to the community from the potential transmission of highly drug-resistant strains harboured by these patients, the DST profiles at the time of treatment failure for the six patients still alive in November 2006 were assessed. Four cases of XDR-TB were recorded at the time treatment was stopped among these 6 patients. A further case was also potentially XDR-TB, but the cultured strain was measured to be rifampicin-sensitive at the time of treatment failure (previously rifampicin-resistant). The remaining treatment failure case, while developing resistance to the second-line injectables kanamycin and capreomycin, did not show resistance to ofloxacin. Thus, among six patients failing treatment and remaining alive, five (83%; 6% of the total started on treatment) were likely to be infected with XDR-TB strains. Overall, five of these six patients were ambulatory and living in the community in November 2006. The remaining patient was being cared for in one of the TB sanatoria, ostensibly in isolation. Reassuringly, no cases of XDR-TB were observed among patients who later defaulted from treatment.

## Discussion

The provision of effective treatment for patients infected with drug-resistant TB is essential, both on humanitarian grounds and if further transmission of such strains is to be prevented. Results from this first cohort of patients treated for MDR-TB in Karakalpakstan, Uzbekistan are promising, with the proportion of successfully treated patients comparable to that seen in other GLC-supported DOTS-Plus projects [5]. Overall, 62% of patients started on treatment were successfully treated. Living in a rural district did not result in poorer treatment outcomes; if anything, rurality tended to improve treatment outcomes, although this did not reach significance. The most common factors mitigating against treatment success (apart from treatment default itself) were, not surprisingly, pre-existing resistance to any second-line anti-TB drugs and the poor clinical condition of patients when started on treatment.

Since this was the first group of patients to be treated in Karakalpakstan, and given the manner in which they were recruited, these patients likely had longer durations of disease and more previous unsuccessful treatment than later patients started on treatment. Such conditions are likely to lead to higher levels of pre-existing drug resistance and poorer clinical condition of patients, which are factors found to be most related to death or treatment failure in this analysis. With the expansion of the capacity of the pilot project to treat more patients in 2004, and given the results of this analysis, there has now been a change in the way MDR-TB is diagnosed in the two districts currently covered by DOTS-Plus. The policy now is for all patients diagnosed with sputum smear-positive TB in these two districts to be routinely tested for drug resistance and be transferred to DOTS-Plus, rather than wait for these patients to fail DOTS treatment regimens. We therefore might expect treatment outcomes to improve in subsequent cohorts. However, the increasing availability of second-line drugs on the open market and the unregulated use of these drugs may lead to even higher levels of second-line resistance than that shown here, with a consequent deleterious impact on treatment success.

In addition to the level of pre-existing second-line drug resistance, the proportion of patients defaulting treatment is also of concern. Overall, 7% of patients found to be infected with MDR-TB strains refused to be treated, and a further 14% of those started on treatment defaulted. Since this was a new treatment program, with a new hospital, anecdotal evidence suggests that initial scepticism may have contributed to patients refusing treatment. Indeed, the proportion of patients declining treatment has significantly reduced over time, particularly when successfully treated patients started to be seen in the community (unpublished observations).

Factors contributing to default from treatment included high alcohol use (assessed at treatment initiation) and the presence of hemoptysis at admission, whereas previous use of second-line drugs was a mitigating factor. Since the use of alcohol was strongly discouraged during treatment, particularly during hospitalisation, and given the high level of alcohol consumption in general in Karakalpakstan, it is not surprising that prior heavy alcohol use was predictive of default. Among subsequent cohorts of patients, much emphasis has been placed on psychosocial care and support for patients, particularly those suffering from alcohol addiction, with the aim of creating a patient-centred approach to care.

The rationale behind the finding that hemoptysis is also related to default is not clear. It is possible that patients consider coughing up blood to be a sign of impending death and consequently feel that treatment will not be successful once this point is reached. Alternatively, patients whose hemoptysis resolves with treatment might consider themselves “cured” and hence stop treatment. Patients who report previous use of second-line drugs appear to be more likely to stay on treatment, while also more likely to be infected with strains with second-line resistance. Previous use of second-line drugs may be a marker for patients who are more committed and motivated to receive and stay on treatment, as these patients have sought out private treatment and paid for these drugs. Or it may be that patients who could afford to purchase second-line drugs in the past have better socioeconomic circumstances and support structures that might mitigate against default. Further qualitative research into the reasons behind default should aid appropriate programmatic responses to reduce the default rate.

The use of an ETR while waiting for DST results did not appear to impact treatment outcomes, based on the absence of significant associations in the univariate and multivariate analyses and on the limited regimen changes occurring when DST results became available. This suggests that empiric regimens (designed according to the prevailing resistance profiles) are an appropriate mechanism for starting suspected MDR-TB patients on treatment earlier. Although a large proportion of patients started on pyrazinamide as part of the ETR were later taken off this drug, its inclusion in the ETR is justified based on its effectiveness among those who might be susceptible. Similarly, the inclusion of ethambutol in the ETR might be justified, given the proportion of patients with strains susceptible to this drug and its relative tolerability and activity against *Mycobacterium tuberculosis*.

The high proportion of patients requiring changes to their treatment regimen, based on both drug resistance and adverse events, suggests that close monitoring and frequent assessment of sputum culture and drug resistance where patients remain or become sputum culture-positive during treatment is essential. The high levels of regimen change and adverse events experienced during treatment reflects the complexity of treatment and the inputs, both medical and laboratory-related, that were required to treat this first cohort of patients. Although the current analysis shows that such treatment is feasible, the level of inputs required reflects the difficulties involved in scaling up drug-resistant TB treatment in settings such as Karakalpakstan.

Scaling up DOTS-Plus should also be tempered by the risk of creating XDR-TB cases. Five patients (6% of the total started on treatment) remained alive, with a high likelihood of continuing to harbour these essentially untreatable TB strains. In this setting, as indeed elsewhere, no further treatment options exist for these patients. Therefore, treatment failure in Karakalpakstan, although at a comparable level to other DOTS-Plus projects, comes at a high cost. To date, no data have been published on the numbers of XDR-TB patients coming out of DOTS-Plus projects. To assess the exact mechanisms and contributors to XDR-TB development, further analyses involving detailed assessment of regimens, compliance and treatment interruptions, along with DNA fingerprinting and testing for mutations contributing to resistance are required. The development of XDR-TB from a project implemented under current best-practice guidelines highlights the urgent need for more effective drugs to treat MDR-TB. In the meantime, strategies to determine the circumstances under which patients with pre-existing XDR-TB should be offered second-line treatment are needed, as is a further discussion of second-line regimens that include all available drugs to which the organism is susceptible to reduce the risk of XDR-TB. In addition, programs need to be responsible for supporting patients who fail treatment with XDR-TB, to reduce the risk of further community transmission.

Since this was a programmatic evaluation, a number of limitations should be considered. The relatively small sample size restricts the scope of analyses that can be conducted. For example, it is not possible to compare treatment outcomes among patients receiving different treatment regimens due to the range of resistance profiles and variety of regimens (with changes throughout treatment) used. Other limitations include the case-finding strategy employed for this first group of patients, resulting in perhaps the most difficult group of patients to treat, along with logistical constraints resulting in delays in treatment initiation, whereby there is more opportunity for strains to become more drug resistant. In addition, the relatively high default rate renders the assessment of treatment efficacy difficult if patients manage to stay on treatment.

In conclusion, these first results from a pilot MDR-TB treatment program in Karakalpakstan, Uzbekistan are acceptable, given the severity of disease and level of drug resistance in the first cohort. These results suggest that treatment in high-prevalence MDR-TB settings is feasible, even in rural areas, albeit resource intensive and difficult for patients and staff. Further simplification and standardisation of regimens would make scaling up treatment more practicable, but potentially increases the risk of XDR-TB development.
